# Research progress on the reduced neural repair ability of aging Schwann cells

**DOI:** 10.3389/fncel.2023.1228282

**Published:** 2023-07-20

**Authors:** Hao Zhang, Zhong Zhang, Haodong Lin

**Affiliations:** Department of Orthopedic Surgery, Shanghai General Hospital, Shanghai Jiao Tong University School of Medicine, Shanghai, China

**Keywords:** peripheral nerve injury, aging, repair Schwann cells, c-Jun, dedifferentiation, neurotrophic factors, myelin clearance, remyelination

## Abstract

Peripheral nerve injury (PNI) is associated with delayed repair of the injured nerves in elderly patients, resulting in loss of nerve function, chronic pain, muscle atrophy, and permanent disability. Therefore, the mechanism underlying the delayed repair of peripheral nerves in aging patients should be investigated. Schwann cells (SCs) play a crucial role in repairing PNI and regulating various nerve-repair genes after injury. SCs also promote peripheral nerve repair through various modalities, including mediating nerve demyelination, secreting neurotrophic factors, establishing Büngner bands, clearing axon and myelin debris, and promoting axon remyelination. However, aged SCs undergo structural and functional changes, leading to demyelination and dedifferentiation disorders, decreased secretion of neurotrophic factors, impaired clearance of axonal and myelin debris, and reduced capacity for axon remyelination. As a result, aged SCs may result in delayed repair of nerves after injury. This review article aimed to examine the mechanism underlying the diminished neural repair ability of aging SCs.

## 1. Introduction

Peripheral nerve injury (PNI) involves the impairment of the peripheral nerve trunk and its ramifications resulting from various factors, such as trauma, tumor, metabolic disease, or iatrogenic intervention, especially external trauma (De la Rosa et al., 2018). Axon and myelin sheath rupture and disintegrate at the site of injury after PNI, leading to the degeneration of distal segment of the nerve, which is known as Wallerian degeneration (Li et al., 2021). The peripheral nervous system (PNS) has a regeneration capability in mammals. Axons regenerate at 1–3 mm daily after PNI to reach the motor endplates and innervate corresponding muscles (Slavin et al., 2021). However, incomplete recovery of axons occurs in about one-third of PNI patients, leading to inadequate restoration of function (loss of sensory and motor functions), chronic pain, muscle atrophy, and even permanent disability (Wang et al., 2019).

Aging is one of the major causes of incomplete recovery of the peripheral nerve (Büttner et al., 2018). The impairment of peripheral nerve repair in elderly patients poses a significant burden on families and society due to a gradual increase in the aging population in industrialized countries and thus requires an urgent solution. Research has demonstrated that age-related changes such as persistent inflammation, delayed macrophage response to injury, Schwann cells (SCs) dysfunction, and changes in the microenvironment reduce the regenerative capability of the PNS in murine models. In aged animals, there are fewer axonal protrusion buds and an altered chromatolytic response in neurons. The distribution of axon microtubules and microfilaments and nerve regeneration at the terminals are also altered (Tanaka et al., 1992). The number of macrophages in sciatic nerves of old mice is significantly increased without injury, indicating a chronic inflammatory microenvironment. In aged mice, macrophage numbers and cytokine markedly decreased on day 3 after injury but were considerably higher at 8 weeks than in young mice, which reflects a delayed acute immune response with a persistent chronic inflammatory response. Because of the delayed macrophage response, the clearance of myelin and chemotaxis of other fundamental molecules for successful Wallerian degeneration is reduced in old mice. The persistence of proinflammatory macrophages and higher cytokine expression also becomes one of the inhibitory elements of nerve regeneration in aged mice. Additionally, SCs dysfunction is an important factor in delayed repair of PNI (Büttner et al., 2018; Maita et al., 2023).

**FIGURE 1:**
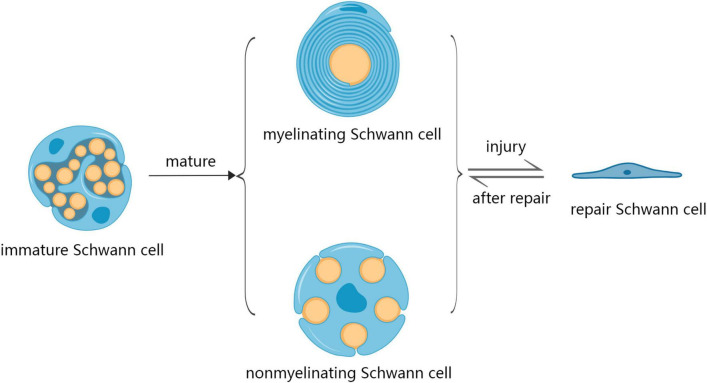
The development process of Schwann cells during development and after injury. SCs mature in two distinct conformations: myelinating Schwann cells (mSC) and non-myelinating Schwann cells (nmSC). SCs acquire a repair phenotype (repair Schwann cells; RSCs) after injury as a regenerative response, after which the repair of nerve RSCs returns into the phenotype of mSCs and nmSCs.

Schwann cells are glial cells found in the PNS involved in repairing injuries to peripheral nerves (Galino et al., 2019). SCs originate from multipotent neural crest precursors, which migrate within nascent peripheral nerves and undergo two distinct conformations: myelinating Schwann cells (mSCs) and non-myelinating Schwann cells (nmSCs) – mSC establishes an exclusive relationship with one axon, while nmSC (Remak Schwann cells) enclose multiple axons of smaller caliber (Monje, 2020; Muppirala et al., 2021). SCs initiate a regenerative response after PNI by dramatically changing the expression of various genes involved in nerve repair. SCs acquire a repair phenotype (Repair Schwann Cells; RSCs) after PNI to promote nerve regeneration and functional recovery (Figure 1; Bolívar et al., 2020). However, SCs undergo various changes during aging that can potentially reduce their repair abilities. For example, SCs aging is associated with decreased cytoplasmic volume occupied by mitochondria and increased residual bodies and myelin debris, which can reduce energy availability and hinder nerve regeneration, leading to delayed functional recovery (Pannese, 2021). Changes in metabolic patterns, reduced stress adaptation ability, accumulation of damaged proteins, lipids and DNA, as well as pathological and traumatic factors can also affect the repair ability of SCs (Sardella-Silva et al., 2021). In an aged environment, SCs exhibit a slow activation of the transcriptional repair system, which results in diminished dedifferentiation, macrophage recruitment, and myelin clearance ability (Maita et al., 2023). Therefore, the mechanisms underlying SCs aging should be evaluated to develop effective strategies for nerve repair after PNI.

Although numerous recent studies have investigated the underlying mechanism of SCs in repairing damage to the PNS. The pathophysiological mechanism underlying delayed nerve repair of aging SCs is unclear. Therefore, further research should elucidate how SCs transform with age and how these changes impact their repair ability. This article aimed to review the relevant literature evaluating the pathophysiological mechanism underlying the decreased neural repair capacity of aging SCs.

## 2. Search strategy

Studies cited in this review were searched on the PubMed database. These studies were published between 1999 and 2022. Literature retrieval was performed using the following keywords: peripheral nerve injury, aging Schwann cell, nerve repair, aging nerve, c-Jun, Schwann cell dedifferentiation, neurotrophic factors, myelin clearance, macrophages, and remyelination. The aim of this review is to explore the mechanisms by which aging SCs cause delayed nerve repair. Therefore, we selected “peripheral nerve injury,” “aging Schwann cell,” “nerve repair,” and “aging nerve” as search keywords. Previous literature has shown that c-Jun functions as a single independent initiator of the repair SC phenotype, which is essential for the dedifferentiation and transdifferentiation of SCs and subsequent regeneration (McMorrow et al., 2022). Therefore, exploring the changes in c-Jun in aging SCs is extremely important. In addition, secretion of neurotrophic factors combined with macrophage clearance of myelin and remyelination are important ways for SCs to play a role in repair (Nocera and Jacob, 2020). Therefore, we also selected “neurotrophic factors,” “myelin clearance,” “macrophages,” and “remyelination” as search keywords. The search was completed on June 2023 (Table 1).

**TABLE 1:** Summary of key papers exploring the aging Schwann cells and nerve repair.

References	Literature number	Type	Findings
Büttner et al., 2018	10.1111/acel.12833	Original research	Identifies CCL11 as a promising target for anti-inflammatory therapies aiming to improve nerve regeneration in old age
Tanaka et al., 1992	10.1016/0014-4886(92)90022-i	Original research	Myelinated fiber regeneration could be retarded by suppressing macrophage responses and was not significantly changed by conditioning lesions before crush injury
Maita et al., 2023	10.1016/j.jss.2023.03.017	Review	The interaction between macrophages and SC plays a crucial role in the nerve regeneration of aged models
Pannese, 2021	10.4081/ejh.2021.3249	Review	The molecular profiles of neuroglia also change in old age, which, in view of the interactions between neurons and neuroglia
Jessen and Mirsky, 2016	10.1113/jp270874	Review	The repair Schwann cell and its function in regenerating nerves
Brosius Lutz et al., 2022	10.1186/s12974-022-02462-6	Original research	Provides a valuable platform for understanding key differences in the PNS and CNS glial responses to injury and for designing approaches to ameliorate CNS regeneration
Welleford et al., 2020	10.1177/0963689720926157	Original research	Provides new insights regarding the essential interactions of different molecular pathways that drive neuronal repair and axonal regeneration in humans
Nocera and Jacob, 2020	10.1007/s00018-020-03516-9	Review	Discusses the main steps of the repair program with a particular focus on the molecular mechanisms that regulate SC plasticity following peripheral nerve injury
Kang and Lichtman, 2013	10.1523/jneurosci.4067-13.2013	Original research	Facilitating clearance of axon debris might be a good target for the treatment of nerve injury in the aged
Fazal et al., 2017	10.1523/jneurosci.0986-17.2017	Original research	Modest c-Jun elevation, which is beneficial for regeneration, is well tolerated during Schwann cell development and in the adult and is compatible with restoration of myelination and nerve function after injury
Painter et al., 2014	10.1016/j.neuron.2014.06.016	Original research	Age-associated decline in axonal regeneration results from diminished Schwann cell plasticity, leading to slower myelin clearance
Sardella-Silva et al., 2021	10.3390/biom11121887	Review	This review gathers essential information about Schwann cells in different stages, summarizing important participation of this intriguing cell in many functions throughout its lifetime
Chen et al., 2017	10.1142/s0218810417500514	Original research	The process of Schwann cell dedifferentiation following peripheral nerve injury shows different trends with age
Wagstaff et al., 2021	10.7554/eLife.62232	Original research	Reduced c-Jun in Schwann cells regulates success and failure of nerve repair both during aging and chronic denervation
Jessen and Arthur-Farraj, 2019	10.1002/glia.23532	Review	Discussed the emerging similarities between the injury response seen in nerves and in other tissues
Jessen and Mirsky, 2021	10.3389/fncel.2021.820216	Original research	Failure of c-Jun expression is implicated in repair cell failures in older animals and during chronic denervation
Scheib and Höke, 2016	10.1016/j.neurobiolaging.2016.05.004	Original research	Both macrophages and Schwann cells had attenuated responses to nerve injury in aged rats, leading to inefficient clearance of debris and impaired axonal regeneration
Shi et al., 2021	10.1016/j.jot.2021.09.004	Original research	RAB7A, ARF6, ARF1, VPS45, RAB11A, DNM3, and NEDD4 were the core markers and may control the molecular mechanism of the endocytosis pathway
Lu et al., 2022	10.1093/bfgp/elab009	Original research	Both molecular and cellular phenotypes of innate immune cells that contribute to age-related inflammation
Cantuti-Castelvetri et al., 2018	10.1126/science.aan4183	Original research	Cholesterol-rich myelin debris can overwhelm the efflux capacity of phagocytes, resulting in a phase transition of cholesterol into crystals and thereby inducing a maladaptive immune response that impedes tissue regeneration
Sakita et al., 2016	10.1186/s12868-016-0277-4	Original research	Both the distal MFs and capillaries in the peripheral nerve may simultaneously regress with aging
Sakita et al., 2018	10.1152/japplphysiol.00257.2018	Original research	Regular, moderate-intensity aerobic exercise may help to prevent and reverse peripheral nerve regression in older adults
Tanaka et al., 1992	10.1016/0014-4886(92)90022-i	Original research	Myelinated fiber regeneration could be retarded by suppressing macrophage responses and was not significantly changed by conditioning lesions before crush injury
Sakita et al., 2020	10.1111/joa.13168	Original research	Myelinated fibers of peripheral nerves show signs of regression in elderly rats
Djuanda et al., 2021	10.1007/s12031-020-01768-5	Original research	Lipid metabolism might play an important role in maintaining the structure and physiological function in sciatic nerves during aging
Shen et al., 2011	10.1016/j.neulet.2011.07.034	Original research	Myelin-associated proteins perform distinct actions on the formation, maturation, degeneration, and regeneration of myelin sheaths
Liu et al., 2018	10.4103/1673-5374.241469	Original research	In peripheral nerves, cells and the microenvironment change with age, thus influencing the function and repair of peripheral nerves
Giorgetti et al., 2019	10.1038/s41598-019-49850-2	Original research	Underlines the power of MTR for the study of peripheral nerve injury in small tissues such as the sciatic nerve of rodents and contributes new knowledge to the effect of aging on recovery after injury
Han et al., 2019	10.4103/1673-5374.253511	Review	Provides a comprehensive basis on which to make clinical decisions for the repair of peripheral nerve injury
Fuertes-Alvarez and Izeta, 2021	10.14336/ad.2020.0708	Review	Summarizes the current knowledge about the implication of tSCs in the age-associated degeneration of NMJs
Schira-Heinen et al., 2022	10.3390/ijms231810311	Original research	Sphingosine-1-phosphate receptors augments a repair mediating Schwann cell phenotype

## 3. Mechanism of SCs promoting peripheral nerve repair

Schwann cells undergo various changes, such as changes in the expression of genes related to nerve repair, after PNI to promote the repair process. Nerve repair after PNI involves two successive and partially overlapping stages. In the initial stage, SCs detach from the distal segment of axons and demyelinate, followed by dedifferentiation and trans-differentiation of SCs into RSCs (Jessen and Mirsky, 2016; Brosius Lutz et al., 2022), thus activating a series of repair functions. Welleford et al. (2020) analyzed the whole transcriptome profile of the human peripheral nerve after injury. They found that genes related to the cell cycle, cell proliferation, immune cell function, synaptic structure, and neuron function revealed significant changes, myelination-related gene transcripts were downregulated and genes related to growth factor activity were upregulated. Besides, SCs transdifferentiated or reprogrammed from a mature form into a repair phenotype. Chau et al. (2022) reached a similar conclusion in a study of transection injury to the human sural nerve.

### 3.1. SCs mediate demyelination of injured nerve

Several pro-myelinating genes, such as *Early Growth Response 2* (*Erg2* or *Krox20*) and other myelin-related genes, including *Myelin Basic Protein* (*MBP*), *Myelin Protein Zero* (*Mpz* or *P0*), *Peripheral Myelin Protein 22* (*Pmp22*), and *Myelin Associated Glycoprotein* (*Mag*) are down-regulated after PNI (Nocera and Jacob, 2020). Furthermore, the upregulation of F-actin within SCs that accumulate at the myelin Schmidt-Lantermann incisures (SLI) occurs after PNI, damaging E-cadherin/catenin complexes, thus leading to the demyelination of injured nerves (Jung et al., 2011; Tricaud and Park, 2017).

### 3.2. SCs dedifferentiate and transdifferentiate into RSCs

Schwann cells undergo dedifferentiation after PNI, leading to a state reminiscent of neonatal SC progenitors, and thus they are converted into RSCs (Xu et al., 2020; Stassart and Woodhoo, 2021). The transcription factor, which rapidly increases 80 to 100-fold after PNI, regulates SCs reprogramming by directly or indirectly modulating the expression of at least 172 genes within SCs post-injury (Arthur-Farraj et al., 2017). These genes are involved in several neural repair processes, including SCs dedifferentiation, myelin clearance, neuronal survival, and axonal regeneration (Norrmén et al., 2018; Della-Flora Nunes et al., 2021). These processes promote nerve repair by triggering the secretion of neurotrophic factors, formation of bands of Büngner, clearance of damaged axons and myelin, and stimulation of axonal remyelination (Glenn and Talbot, 2013; Fornaro et al., 2021).

Repair Schwann cells secrete various neurotrophic factors involved in the repair process after PNI, including nerve growth factor (NGF), brain-derived neurotrophic factor (BDNF), glial cell line-derived neurotrophic factor (GDNF), neurotrophin-3 (NT-3), neurotrophin-4/5 (NT-4/5), ciliary neurotrophic factor (CNTF), and fibroblast growth factor (FGF) (Fontana et al., 2012; Huang et al., 2019).

The accumulation of axon and myelin debris at the site of nerve injury can impede nerve repair. SCs play a critical role in accelerating the clearance of debris. SCs can phagocytose axonal debris and digest myelin after injury, establishing a favorable environment for neural repair and axon regeneration (Vaquié et al., 2019; Wang et al., 2020). Myelin clearance involves intracellular and extracellular components. For intracellular components, RSCs can clear debris through autophagic destruction (myelinophagy) (Reed et al., 2020). RSCs can initiate myelin breakdown, attracting macrophages for debris phagocytosis (extracellular component) (Gomez-Sanchez et al., 2015). RSCs up-regulate several cytokines, such as tumor necrosis factor α (TNFα), interleukin-1α (IL-1α), IL-1β, leukemia inhibitory factor (LIF), monocyte chemotactic protein 1 (MCP-1), and fibroblast growth factor 9 (FGF9), during myelin clearance to activate the innate immune response (Jessen and Mirsky, 2016; Qu et al., 2021).

Repair Schwann cells are shortened by about sevenfold after conversion from the repair phenotype to the myelin phenotype, thereby generating typically short internodes in regenerated nerves (Gomez-Sanchez et al., 2017). RSCs up-regulate myelin proteins, including neuregulin-1 (NGR-1) type III, adhesion G protein-coupled receptor (GPCR), and tyrosine kinase (Fyn) after reconnecting with axons through activation of multiple signaling pathways, including Par-3/Par-6/aPKC, PI3K/Akt/mTOR, Ga6-Tyro3, NRG-1/ErbB2/3 (Birchmeier and Bennett, 2016; Torii et al., 2019). As a result, RSCs redifferentiate into mSCs, facilitating remyelination of large-caliber axons, while Remak SCs stimulate ensheathing of small-caliber fibers to reform Remak bundles (Ulrichsen et al., 2022).

## 4. Mechanism of aging SCs delaying peripheral nerve repair

**FIGURE 2:**
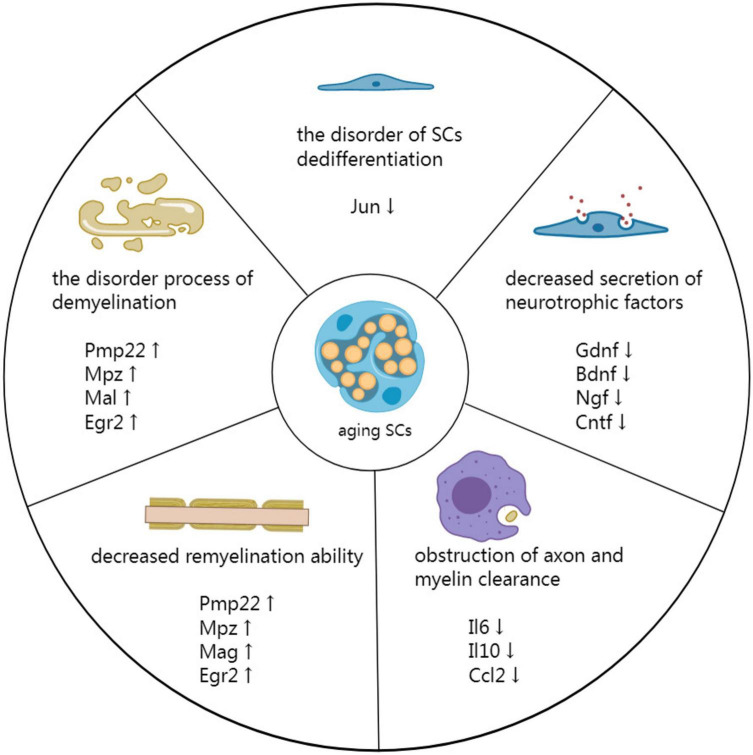
Mechanism by which aging SCs delay peripheral nerve repair. Demyelination is a disorderly process associated with impeded PNI repair. Additionally, disorders involving SC dedifferentiation have detrimental effects on the repair process. The secretion of neurotrophic factors is reduced in aging SCs, affecting all phases of nerve repair. Moreover, the reduced secretion affects the clearing of axons and myelin by SCs and macrophages at the distal end of injured nerves. Finally, decreased remyelination ability of aging SCs results in inefficient remyelination of new axons.

The diminished repair ability of aging SCs is associated with impaired nerve repair after PNI in older animals (Kang and Lichtman, 2013). Recent studies have shown that several factors promote delayed nerve repair in aging SCs, including demyelination and dedifferentiation disorders, decreased secretion of neurotrophic factors, obstruction of axon and myelin clearance, and reduced remyelination ability (Figure 2; Fazal et al., 2017).

### 4.1. Demyelination and dedifferentiation disorders associated with aging SCs

Genes related to myelin production in aging SCs, such as *Pmp22*, *Mpz*, *Mal*, and *Egr2* are overexpressed after PNI. As a result, these genes have decreased myelinating ability, leading to delayed demyelination of injured nerves (Painter et al., 2014). Demyelination is the first step involved in nerve repair after PNI, and thus delayed demyelination can delay subsequent physiological processes, ultimately impeding the repair of injured nerve.

Additionally, delayed demyelination weakens the ability of aging SCs to transdifferentiate into RSCs, thus limiting nerve repair. Significant downregulation of mitosis-related genes (*Kif2c*, *Pbk*, *Birc5*, and *Cdc20*) and growth factor genes (*Btc*, *Ngfr*, and *Bdnf)* can also delay demyelination (Painter et al., 2014; Sardella-Silva et al., 2021). The reduced neural repair ability of aging SCs is caused by the failure to maintain or activate high levels of the transcription factor c-Jun, which promotes SC dedifferentiation. c-Jun also acts as a global amplifier of the RSC phenotype (Chen et al., 2017; Wagstaff et al., 2021). Wagstaff et al. (2021) reported that c-Jun protein levels within the distal nerve stump are about 50% lower in aged mice than in young mice on the fourth day after PNI. Furthermore, increased c-Jun levels in SCs reversed age-related defects in nerve regeneration. As a result, the same number of regenerated neurons and CGRP+ nerve fibers were similar between the older and younger animals. Painter et al. (2014) also reported that c-Jun protein levels in the nerves are significantly different between the aged and young animals immediately after injury. c-Jun expression was significantly higher in the nerves of young animals 1 day after injury (about fivefold higher) than in aged animals. In an analysis involving 173 c-Jun-regulated injury genes, 138 genes were differentially expressed between young and aged mice in both uninjured and injured nerves. c-Jun down-regulation inhibits the expression of multiple genes closely related to SC proliferation and differentiation, including *Gpr37L1*, *Igfbp2*, and *Olig1* (Boerboom et al., 2017; Wagstaff et al., 2021). As a result, the repair ability of SCs is inhibited and neural cell adhesion molecule (NCAM), p75 neurotrophin receptor (p75^NTR^), and glial fibrillary acidic protein (GFAP) (Jessen and Arthur-Farraj, 2019) are down-regulated, thus delaying the repair of injured nerves.

### 4.2. Aging SCs are associated with decreased secretion of neurotrophic factors

Decreased secretion of neurotrophic factors after injury also affects the repair capacity of aged SCs. Besides, recent research has elucidated that aging SCs cannot efficiently secrete various neurotrophic factors, such as GDNF, BDNF, NGF, CNTF, NT-3, and β-cellulin (Btc) (Jessen and Mirsky, 2021).

Glial cell line-derived neurotrophic factor can promote the survival of motor neurons, neurite outgrowth, myelination, and remodeling of neuromuscular junctions (Cintron-Colon et al., 2022). GDNF down-regulation in aging SCs delays neural repair. BDNF is rapidly up-regulated in the early phase of nerve injury and mediates early nerve repair. BDNF has two receptors, tropomyosin receptor kinase B (trkB) and p75^NTR^. The BDNF/trkB signaling pathway promotes the transport of actin filaments and associated proteins toward the growth cone, thereby promoting axon outgrowth. p75^NTR^ mediates remyelination through a BDNF-dependent mechanism. p75^NTR^ down-regulation delays functional recovery and axonal growth (McGregor and English, 2018). Overall, BDNF down-regulation in aging SCs delays neural repair. Additionally, NGF can enhance autophagic activities in SCs via the p75NTR/AMPK/mTOR dependent pathways, thus promoting the clearance of axons and myelin in the early stage of PNI (Li et al., 2020). CNTF mediates a neuroimmune cascade by activating signal transducer and activator of transcription 3 (STAT3) and inducing interleukin 6 (IL-6) (Hu et al., 2020), thus promoting the survival of nerve cells and the regeneration of axons through the ERK1/2/MAPK pathway (Fan et al., 2017). Other neurotrophic factors, such as NT-3, are also involved in nerve repair. Unlike BDNF/p75NTR, which inhibits SC migration and enhances myelin formation, NT-3/TrkC promotes SCs migration while inhibiting myelin formation. NT-3 also mediates the activation of downstream MAKP/ERK and AKT signaling pathways, thus facilitating *in vitro* SC migration. NT-3 down-regulation can decrease the myelination of regenerating axons. Furthermore, NT-3/TrkC participates in the regeneration of Remak bundles (Ulrichsen et al., 2022), indicating that reduced NT-3 levels in aging SCs can significantly delay nerve regeneration after PNI. SCs-secreted Btc is significantly up-regulated in injured segments, driving SCs proliferation, thus stimulating their migration, regulating their phenotype, influencing neuron behavior, increasing neurite length, and mediating nerve regeneration. In one study (Wang et al., 2021), the scratch area of SCs transfected with siRNA control decreased by nearly twofold compared with the scratch area SCs transfected with siRNA against Btc, consistent with transwell migration assay. Furthermore, neurons co-cultured with SCs transfected with siRNA control had longer neurites than those with siRNA against Btc. These assays suggest that Btc is essential after PNI.

Therefore, down-regulation of the above neurotrophic factors secreted by SCs can affect the stages of nerve repair, thus delaying PNI-related repair.

### 4.3. Aging SCs are associated with obstruction of axon and myelin clearance

Senescent SCs have decreased ability to assimilate and digest axons and myelin sheaths at the distal end of injured nerves, limiting Wallerian degeneration in aged nerves. Therefore, inefficient removal of axons and myelin sheaths inhibits axon extension (Scheib and Höke, 2016). Painter et al. (2014) showed that axons in the nerve distal to the injury are degenerated after PNI and cleared. Although myelin sheaths are degenerated in young mice within 3 days post-PNI, aged mice have more axons with intact myelin sheaths (about four times) than young mice, indicating that myelin and axon clearance is delayed in aging mice. Scheib and Höke (2016) also showed that myelin debris increases in distal-aged rats. They also showed that aged grafts in aged rats had more debris than young grafts in young rats, indicating that age affects debris clearance. Moreover, Painter et al. (2014) revealed that phagocytosis of myelin is decreased by 35% in aged compared with young SCs based on Florescent-activated cell sorting, which was not attributable to reduced viability of aged SCs. Scheib and Höke (2016) demonstrated that SCs from young rats engulf more myelin than those from aged rats, indicating that SCs from young rats are more phagocytic than those from aged rats. The SCs aging is associated with gradual decrease in various autophagy functions, thus obstructing axon and myelin clearance (Longo et al., 2015; Shi et al., 2021).

Furthermore, aging SCs secrete fewer cytokines, impeding prompt recruitment of macrophages, further hindering clearance of damaged axons and myelin. The expression of cytokines, such as pro-inflammatory IL-6, anti-inflammatory IL-10, and arginase-1, is decreased in aging nerves, slowing down macrophage recruitment at the injury site and reducing clearance of axons and myelin. The secretion of CCL2, a key chemoattractant for macrophages, s decreased in aging SCs (Scheib and Höke, 2016). Phagocytosis assays have indicated that macrophages from young rats are more phagocytic than those from aged ones. Although there are many macrophages in aged rats, only a few may infiltrate the injury site after PNI. More macrophages enter young nerve grafts in young rats than aged grafts in aged rats (Scheib and Höke, 2016; Lu et al., 2022). Cantuti-Castelvetri et al. (2018) also reported that myelin debris, lipid droplets, and needle-shaped cholesterol crystals accumulate in phagocyte lysosomes in old mice, a typical hallmark of cholesterol overloading found in numerous foam cells. Lesion restitution failure in old mice could be due to the inability to clear excessive myelin-derived cholesterol from phagocytes.

In conclusion, impaired phagocytosis and decreased cytokine secretion in aging SCs affect axon and myelin clearance, resulting in physical blockage of regenerated axons, thus slowing the repair of damaged nerves.

### 4.4. Aging SCs are associated with decreased remyelination ability

Schwann cells aging causes myelination abnormalities in mice. Furthermore, the number of myelinated nerve fibers is significantly decreased in older mice. Myelinated fiber diameter, axon perimeter, myelin thickness, and myelin perimeter are also significantly decreased in older mice compared with those in younger mice (Sakita et al., 2016, 2018). Tanaka et al. (1992) reported that after crush injury, nerves from aging mice contained significantly fewer regenerating myelinated fibers, with smaller axons and thinner myelin sheaths. The length and thickness of the myelin sheath are controlled by axolemmal “myelinating” signals and their receptors. Aging SCs may have an abnormal number or distribution of receptors, which delays remyelination of regenerating axons (Tanaka et al., 1992). Sakita et al. (2020) also reported that the structure of myelin sheaths and axons in myelinated fibers is altered in aged mice, with many irregularities stemming from degeneration. Furthermore, they showed that the mean fiber diameter, axon diameter, and myelin thickness are significantly lower in the aged group than in the young and middle-aged groups. Moreover, aging mice have tiny vacuoles within the myelin sheath, accompanied by thinning and some dislodged myelin walls. Fiber loss and massive disorganization also occur within the myelin sheath of aging mice, culminating in irregular disintegrated and dysmyelinated fibers (Djuanda et al., 2021). These changes are mainly caused by down-regulation of myelin proteins. Djuanda et al. (2021) also showed that MBP is significantly down-regulated in healthy aging nerves at 6 months, which remained low until the late stages of life. Other myelin proteins, such as MAG, MPZ, and PMP22, are also significantly down-regulated in aged SCs (Shen et al., 2011).

Furthermore, aging SCs lead to inefficient remyelination of newborn axons. Myelin-associated proteins, such as PMP22, MPZ, MAG, and EGR2 are down-regulated in aged SCs during the myelin repair stage after PNI, reducing remyelination ability (Liu et al., 2018). Giorgetti et al. (2019) showed that myelin and axons are highly damaged and disorganized in both young and aging mice, with the ring structure of myelin disappearing and intense myelin accumulation occurring at week 1 after injury. Although the young mice fully recovered by week 6, the level of myelin in older mice remained low, ultimately delaying nerve repair in older mice. Additionally, myelin trophic factors, such as prosaposin (PSAP) and prosaptide are secreted after PNI, thus promoting sulfide synthesis in the myelin sheath, mRNA expression of MPZ, and UDP-galactose expression (ceramide galactosyltransferase; GalT), thereby promoting repair of myelinated nerves (Hiraiwa et al., 1999). Although G protein-coupled receptors GPR37 and GPR37L1 serve as receptors for PSAP and prosaptide (Taniguchi et al., 2021; Massimi et al., 2022), the expression of these receptors is low in aging SCs (Wagstaff et al., 2021), leading to peripheral nerve remyelination disorders and delayed nerve repair. Cantuti-Castelvetri et al. (2018) indicated that inadequate clearance of damaged myelin results in the accumulation of cholesterol crystals, eventually impairing remyelination. Cholesterol crystals can induce inflammation by phagolysosomal membrane rupture, followed by stimulation of the caspase-1-activating NLRP3 (NALP3 or cryopyrin) inflammasome and secretion of IL-1 cytokine, thus activating NLRP3 inflammasome in macrophages. Uncontrolled inflammation impairs inflammation resolution and subsequent repair processes in aging animals, thus delaying remyelination (Cantuti-Castelvetri et al., 2018).

## 5. Conclusion

Peripheral nerve injury induces protracted delays in the repair of nerve damage among elderly patients. The morphological, physiological, and biochemical transformations caused by the aging of peripheral nerves affect the efficacy of regeneration. Therefore, the neural repair impairment mechanism in aging individuals should be evaluated due to the increasing human longevity and the number of elderly citizens. SCs are crucial in the developmental and regenerative responses of peripheral nerves. Therefore, understanding SCs is crucial for exploring homeostasis in peripheral nerves and regeneration mechanisms. Furthermore, the mechanism underlying the delayed repair of PNI should be investigated. The role of senescent SCs in the restoration of damaged nerves should also be analyzed.

Several studies have examined the pathophysiological mechanism of aging SCs and their impact on delayed nerve repair. This review focused on the principal mechanisms underlying the diminished neural repair capabilities of aging SCs in the latest research. The disorderly process of demyelination affects the expression of various myelin genes in senescent SCs, thus impeding the repair process of PNI. Additionally, disorderly dedifferentiation of SCs further delays the repair process, primarily due to c-Jun down-regulation. Furthermore, aging SCs reduce the secretion of neurotrophic factors, including GDNF, BDNF, NGF, CNTF, NT-3, and Btc, thus affecting all stages of nerve repair. Moreover, SCs and macrophages cannot efficiently clear axons and myelin at the distal end of injured nerves, thus affecting the physical regeneration of axons and resulting in uncontrolled inflammation that hinders remyelination. Senescent SCs mediate myelination irregularities, which cause inefficient remyelination of nascent axons.

Nonetheless, a comprehensive network of mechanisms by which aging SCs delay neural repair after PNI should be evaluated. Furthermore, the pathophysiology of aging SCs should be evaluated. Numerous studies have been conducted on therapeutic strategies for aging cells. These include conventional senotherapeutics, prodrugs, protein degraders, nanocarriers, and immunotherapies. The most common senotherapeutic strategy is selectively killing aging cells, known as senolytics. Another way is senomorphics, which reduces the detrimental effects of the senescence-associated secretory phenotype (SASP). In addition, senoreverters, galactose-based prodrugs, proteolysis-targeting chimera, nanocarriers, immunotherapy based on the senescent cell surfaceome are all important therapeutic strategies for aging cells (Birch and Gil, 2020; Zhang et al., 2022). Potential therapies against aging SCs have been proposed, such as maintenance of c-Jun levels to reverse age-related defects, transplantation of SCs and use of sphingosine-1-phosphate receptor agonist Fingolimod to enhance the repair phenotype (Han et al., 2019; Fuertes-Alvarez and Izeta, 2021; Wagstaff et al., 2021; Schira-Heinen et al., 2022). However, there are still few therapeutic strategies for aging SCs. Therefore, gaining insight into the biology of aging SCs may provide new research directions and therapeutic strategies for preventing and repairing PNI among older people.

## Author contributions

HZ and HL: review design. HZ and ZZ: data collection. HZ, ZZ, and HL: manuscript draft and revision. All authors approved the final version of the manuscript.
